# The MK2 pathway is linked to G-CSF, cytokine production and metastasis in gastric cancer: a novel intercorrelation analysis approach

**DOI:** 10.1186/s12967-020-02294-z

**Published:** 2020-03-26

**Authors:** Fares Qeadan, Pranshu Bansal, Joshua A. Hanson, Ellen J. Beswick

**Affiliations:** 1 Department of Family and Preventative Medicine, University of Utah, Salt Lake City, UT USA; 2 New Mexico Oncology Hematology Consultants, Albuquerque, NM USA; 3 Department of Pathology, University of New Mexico Health Sciences Center, Albuquerque, NM USA; 4 Division of Gastroenterology, Department of Internal Medicine, University of Utah, Salt Lake City, UT USA

**Keywords:** Gastric cancer, Map kinase-activated protein kinase 2, Granulocyte colony-stimulating factor, Cytokines, Chemokines

## Abstract

**Background:**

Gastric cancer is associated with chronic inflammation, but there is still much to understand about the tumor microenvironment and the underlying tumor-promoting mechanisms. The Map kinase-activated protein kinase 2 (MK2) pathway is a regulator of inflammatory cytokine production that we have been studying in gastrointestinal cancers. Here, we set out to determine the significance of this gene in gastric cancer along with its downstream mediators and if there were differences in the primary tumors with and without metastasis.

**Methods:**

Human gastric cancer tissues with and without metastasis were examined for MK2 expression and cytokine profile in organ culture supernatants. Advanced statistical methods including a lower triangular correlation matrix, novel rooted correlation network, linear and logistic regression modeling along with Kruskal–Wallis testing with Sidak correction for multiple testing were applied to gain understanding of cytokines/chemokines linked to metastasis.

**Results:**

The MK2 pathway is strongly linked with metastasis and a panel of cytokines. Gene expression was able to classify gastric cancer metastasis 85.7% of the time. A significant association with a panel of cytokines was found, including G-CSF, GM-CSF, Mip-1β, IFN-α, MCP-1, IL-1β, IL-6, and TNF-α. Mip-1β was found to have the strongest association with MK2 and metastasis after Sidak correction for multiple testing.

**Conclusions:**

MK2 gene expression and a novel associated cytokine panel are linked to gastric cancer metastasis. G-CSF is the strongest cytokine to differentiate between metastasis and non-metastasis patients and had the lowest P value, while Mip-1β showed the strongest association with MK2 and metastasis after Sidak correction. MK2 and associated cytokines are potential biomarkers for gastric cancer metastasis. The novel intercorrelation analysis approach is a promising method for understanding the complex nature of cytokine/chemokine regulation and links to disease outcome.

## Background

Gastric cancer is often diagnosed in later stages with high mortality rates associated with progression [1]. Thus, treatment options for gastric cancer are also more limited than many other cancers making it a more difficult cancer to treat. Gastric cancer is often associated with chronic inflammation and history of *Helicobacter pylori* infection. Chronic inflammation has long been accepted as a risk factor for cancer development and progression, yet new treatment options targeting inflammation in cancer still remain limited. Thus, it is critical to understand the inflammatory pathways linked with more severe disease and poor outcome in order to identify inflammatory biomarkers in order to develop more effective treatment approaches.

We have been studying the Map kinase-activated protein kinase 2 (MK2) pathway as a potential target for inflammation and tumor growth in gastrointestinal cancers in mouse models [2, 3], and here we examine the relevance of this pathway to human gastric cancer. MK2 is downstream of p38 MAP-kinase and is associated with DNA damage and regulation of inflammatory cytokine production, specifically, IL-1β, IL-6, and TNF-α [3–5]. These cytokines are known to have pro-tumorigenic properties. IL-1β polymorphisms are linked with increased gastric cancer risk in humans [6], and in mice, IL-1β overexpression induced gastric inflammation and cancer [7]. IL-6 has been shown to induce gastric tumor cell invasion and is associated with metastasis [8, 9]. Finally, TNF-α production induced by *H. pylori* infection may promote gastric cancer [10, 11] along with TNF-α polymorphisms may also increase risk of developing gastric cancer [12]. Thus, MK2-dowstream cytokines are thought to be important players in chronic inflammation that promotes gastric cancer.

Although IL-1β, IL-6, and TNF-α have been shown to be regulated by MK2 signaling, cytokines often act in autocrine or paracrine manners to regulate production of other cytokines in the tumor microenvironment. We also have recently shown that MK2 regulates chemokine production in mouse models of gastrointestinal cancers [13], suggesting that MK2 may regulate expression of a wider network of cytokines and chemokines than previously thought. The goal of this study is to examine the importance of MK2 in gastric cancer and how MK2 is linked with a broader cytokine/chemokine network than originally shown in the literature. Cytokines are often shown to be associated with cancer risk and prognosis, but often viewed independently. Due to the complexity of cytokine/chemokine regulation, here we have explored more in depth approaches to analyzing a larger panel and their association with the MK2 pathway. Rather than independent markers, we examined how cytokines and chemokines are associated with one another. We found MK2 expression to be linked to gastric cancer metastasis and nine significant cytokine associations, including MK2-dowstream cytokines, IL-1β, IL-6, and TNFα along with other previously unrecognized cytokines linked to MK2; G-CSF, GM-CSF, Mip-1β, IFN-α, MCP-1, and IL-2. VEGF, Mip-1α, and IL-8 were close to reaching significance. MK2 and the associated cytokine network could be a biomarker panel for gastric cancer and MK2 inhibition a potential therapeutic target for gastric cancer.

## Methods

### Human tissue samples

Human tissue samples were collected under an IRB approved human protocol at University of New Mexico Health Sciences Center with the assistance of the UNM Cancer Center Human Tissue Repository. Fresh samples were collected as matched tumor and normal tissues as determined by surgical pathology and transferred to the Beswick lab for processing. Tissue samples were divided into pieces for RNA extraction and cytokine assays.

### Gene expression

RNA was extracted from tissue pieces using a trizol (ThermoFisher Scientific) method according to manufacturer’s instructions. RNA concentrations were measured using a Nanodrop instrument (ThermoFisher Scientific). Real-time PCR was performed according to Applied Biosystems’ two-step protocol. The RT reaction mixture includes random 2.5 μM hexamers, 500 μM dNTPs, 0.4 U/μL of the RNase inhibitors, 5.5 mM MgCl_2_, MultiScribe Reverse Transcriptase (3.125 U/μL) and its buffer, and 1 μg of cellular RNA. The RT step was performed according to the following protocol: 10 min at 25 °C, 60 min at 37 °C, 5 min at 95 °C. Obtained cDNA samples were stored at − 80 °C and used for the PCR reaction step. The PCR reaction mix was prepared using the Assays-on-Demand™ gene expression assay mix (Applied Biosystems) for human 18S and MK2. cDNA (2 µl) was added to a 20 × mix of PCR primers and TaqMan^®^ FAM dye-labeled probe. The reaction was carried out according to the following protocol: 2 min at 50 °C, 10 min at 95 °C (1 cycle), and 15 s at 95 °C and one min at 60 °C (45 cycles) on Applied Biosystem’s StepOnePlus instrument. The endpoint used in real-time PCR quantification, CT, was defined as the PCR cycle number that crossed the signal threshold. Quantification of cytokine gene expression was performed using the comparative CT method and reported as the fold difference relative to 18S mRNA.

### Cytokine arrays

Normal and tumor tissue pieces were divided into 8 mg (± 0.5 mg) sections and incubated in RPMI complete media for 16 h. Supernatants were analyzed by multiplex bead array for 26 cytokines/growth factors (Millipore, Billerica, MA) according to manufacturer’s instructions and analyzed on a Luminex 200 machine.

### Statistical analysis

MK2 and cytokines were described numerically using the median and interquartile range, from the first to the third quartile, for both being much less sensitive to outliers and non-normality. Graphically, we used (i) raw data (with jitter) overlaid with Box-plots, and (ii) Histograms with density curves overlay. The Kruskal–Wallis test was used to test for an overall difference in median levels of MK2 or cytokines between groups while using the Sidak adjustment for multiple testing [14]. To assess effect size for the difference between medians, we used the ratio *A/1*—*A* from the *A* measure of stochastic superiority, which gives the odds that an individual in one group will score higher than an individual in the other group. Simple logistic regression was used to assess the odds of developing metastasis associated with an increase in MK2 utilizing the odds ratio (OR) and its associated 95% confidence interval. The logistic regression model was assessed using the Hosmer and Lemeshow goodness of fit test and model discrimination using the area under a receiver operator characteristic (ROC) curve (AUC) [15]. Simple linear regression was used to evaluate the association between MK2, dependent variables, and each of the cytokines as independent variables, while employing the Sidak adjustment for multiple testing. The Spearman correlation with Sidak adjustment was used to evaluate the intercorrelation between cytokines and MK2 and visualized via a lower triangular correlation matrix and a rooted correlation network [16]. A lower triangular correlation matrix was used to indicate the significance in association between biomarkers from non-metastasis to metastasis. To better understand how MK2 is linked to metastasis in gastric cancer through its interaction with cytokines and chemokines in the tumor microenvironment, we anchor a rooted correlation network on MK2 and firstly connect the root with the most significant markers (i.e. those colored in red or yellow from the lower triangular correlation matrix). Secondly, we connect those markers, which were linked to MK2 in the first step, with other markers that are significantly associated with them in the metastasis state. Repeating this process multiple times until exhausting all subsequent significant associations will complete the rooted correlation network.

## Results

### MK2 gene expression is upregulated in gastric cancer and associated with metastasis

**Fig. 1:**
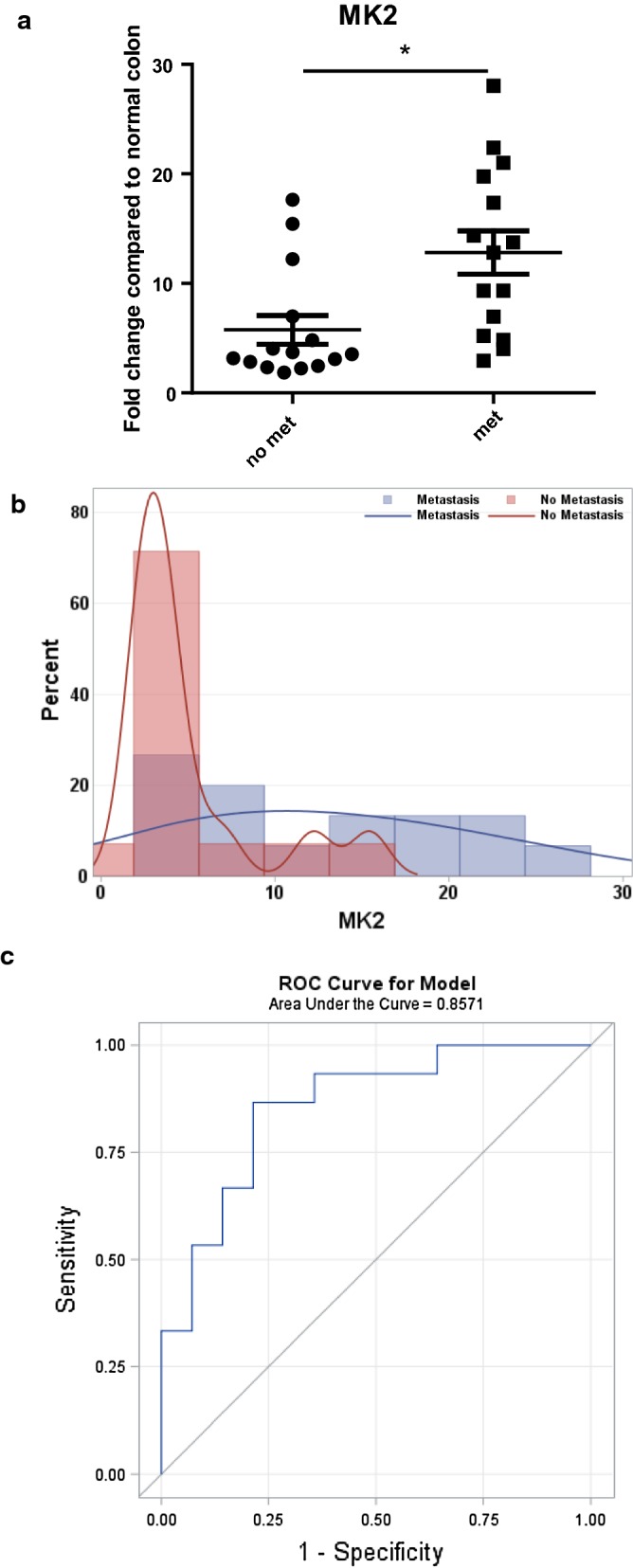
The MK2 pathway is associated with gastric cancer metastasis. **a** MK2 gene expression is significantly increased with gastric cancer lymph node metastasis illustrated via box-plots. **b** an overlay of the distribution of MK2 gene expression fold increase among subjects with and without metastasis. **c** The area under the ROC curve for the association between the odds of developing metastasis within 5 years after surgical resection for every 5 units increase in MK2

Since we found in mouse models that MK2 activation is increased by inflammatory cytokine production [3], here we examined gene expression in a panel of gastric cancer samples. MK2 gene expression was examined by quantitative Real Time (qRT) PCR and scrutinized according tumor stage. While we found a significant increase in T stage 3, the most compelling analysis was with samples that had lymph node metastasis compared to those that did not 5 years after surgical resection (Fig. 1a). Figure 1b further shows the breakdown between metastasis vs non-metastasis samples indicating high elevation in median values of MK2 among those who metastasized (M = 12.81 for those who had metastasis versus M = 3.36 for those who didn’t have metastasis). When data were analyzed using Kruskal–Wallis test, the *p* value was 0.001 (Table 1), suggesting strongly significant gene expression associated with metastasis. In fact, we found that an increase of 5 units of MK2 is statistically significantly associated with an increase of 230.5% in the odds of developing metastasis (OR = 3.305; 95% confidence interval, 1.287–8.482); a large effect with excellent fit (p-value = 0.7977 from the Hosmer and Lemeshow Goodness-of-Fit Test) and high classification power according to the AUC of 0.8571 shown in Fig. 1c. Thus, our data supports a strongly significant association of MK2 gene upregulation with gastric cancer metastasis.

**Table 1:** MK2 median comparison between subjects with Metastasis and those without Metastasis; showing MK2 is significantly increased with metastasis

	MK2 Median (Q_1_, Q_3_)	p-value Kruskal–Wallis test
With metastasis	12.81 (5.21–19.76)	0.001
Without metastasis	3.36 (2.45–4.81)	

### MK2 downstream cytokines are produced in gastric tumors and associated with metastasis

**Fig. 2:**
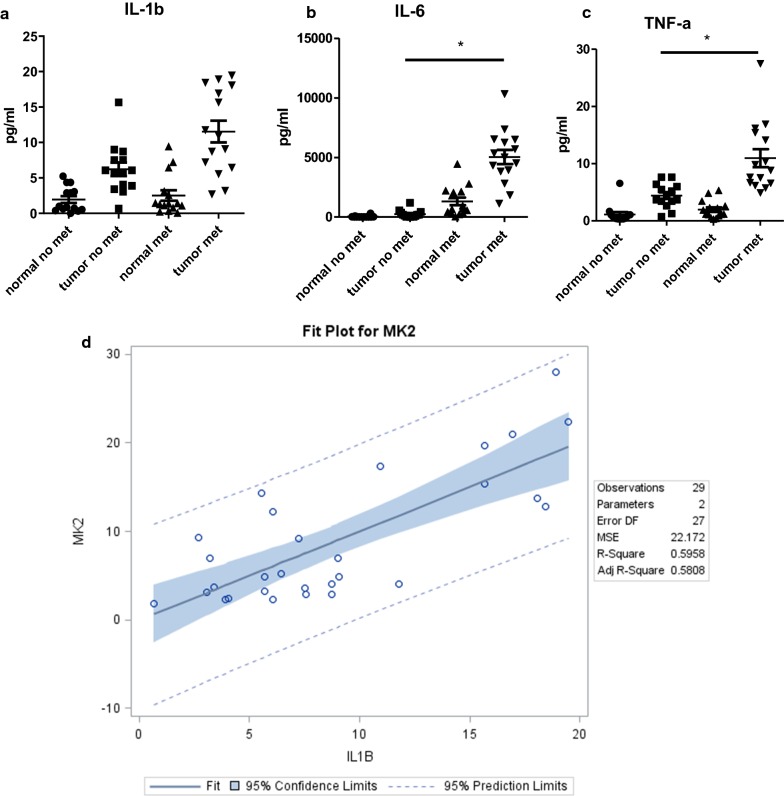
MK2 regulated cytokines **a** IL-1β, **b** IL-6, and **c** TNF-α are increased in tumor tissues compared to matched normal tissues and are significantly higher in primary tumors that have lymph node metastasis and **d** MK2 and IL-1β have a strong linear association. N = 15 for each group

The MK2 pathway is known to be associated with IL-1β, IL-6, and TNF-α production, but may not be recognized for its overall regulation of inflammation in general. Thus, first we examine the cytokines that the MK2 pathway is known to regulate in tissue supernatants. This is an approach we have developed from fresh tissues in our mouse studies [2, 13, 17], and here we have extended this approach to human tissues. We compared normal and tumor tissues for MK2 downstream cytokine production and also found a significant increase in IL-1β, IL-6, and TNF-α (Fig. 2a–c) in tumors with lymph node metastasis compared to non-metastatic tumors. All tumors were increased in production of these cytokines compared matched normal tissues. Of particular interest is that we found IL-1β to be highly correlated with MK2 expression (Fig. 2d), suggesting this cytokine in particular to be critical in association with MK2 expression and subsequently gastric cancer metastasis. Specifically, we found that about 60% of the variability in MK2 could be accounted for by IL-1β alone.

### Other significant cytokine/chemokine production in gastric cancer

**Fig. 3:**
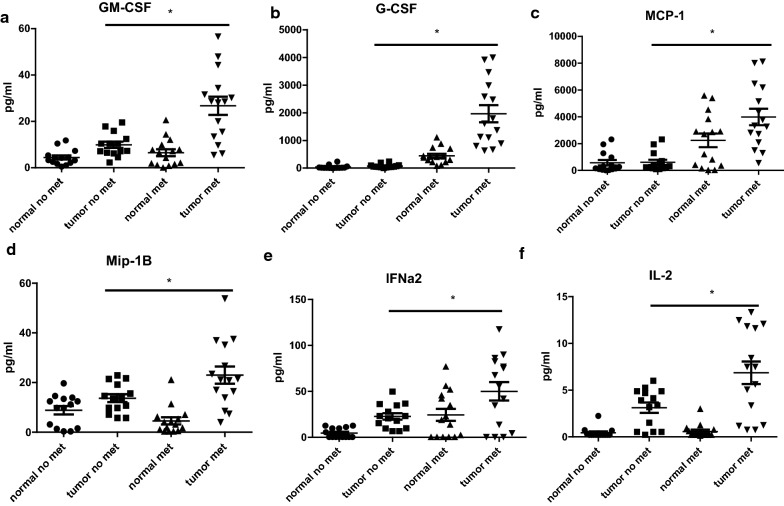
Cytokines previously unknown to be associated with MK2 expression **a** GM-CSF, **b** G-CSF, **c** MCP-1, **d** Mip-1β, **e** IFN-α2, and **f** IL-2 are increased in tumor tissues compared to matched normal tissues and are significantly higher in primary tumors that have lymph node metastasis. N = 15 for each group

**Fig. 4:**
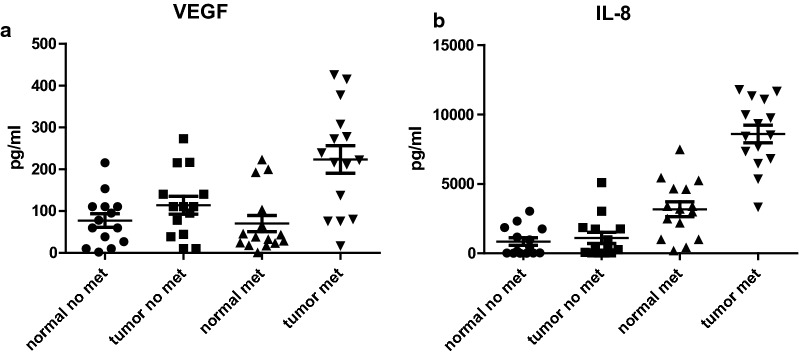
Cytokines/chemokines that are close to significance, but my show effect size; **a** VEGF, and **b** IL-8

Our studies and others have shown an important role for MK2 in the tumor microenvironment [2, 3, 13]. Cytokine/chemokine regulation of other cytokines/chemokines is generally believed to be complex, particularly in inflammation. However, traditional methods of marker comparisons have been examined solely on concentration. Here, we explore a more complex analysis of the intricate network of cytokine expression in gastric cancer and the link to MK2 expression and how this network is linked to metastasis. We found 9 cytokines, out of 26, significantly associated with MK2 expression (Table 2). Among these cytokines were the known MK2 downstream cytokines, IL-1β, IL-6, and TNF-α. Furthermore, macrophage chemotaxis and function cytokines/chemokines were significantly associated with MK2 expression, GM-CSF and MCP-1. Mip-1α and VEGF correlations were close to reaching significance based on p < 0.05. Also of interest is G-CSF, a key cytokine that is highly expressed in human gastric and colon cancers in our previous work [17, 18]. As shown in Table 3, G-CSF was found to be the most statistically significant cytokine to differentiate between metastasized subjects and non-metastasized, followed by IL-6 and IL-8, and other cytokines and chemokines including MCP-1, IL-10, and TNF-α. We note that GM-CSF and Mip-1α were on the boundary of statistical significance and mention that although IL-1β, Mip-1β, and VEGF didn’t show statistical significance difference between metastasized subjects and non-metastasized ones after the Sidak adjustment for multiple testing, their effect size was large enough (i.e. A/1-A > 2.5) to be clinically important. These data suggest an unrecognized role of MK2 in multiple cytokines produced in gastric cancer. Figure 3a–f illustrates their significant association with metastasis, while Fig. 4a, b illustrate cytokines/chemokines that are close to significance, but may show clinically relevant effect size. Furthermore, these data suggest a need to consider not only p values, but also that effect size may be another critical analysis method that should be considered as well.

**Table 2:** Linear association between cytokines and MK2 in 30 subjects (15 with metastasis and 15 without metastasis)

	Analytes*	$\hat{\beta }$	T-stat	p-value**	r	R^2^
1	*IL1B*	*1.00825*	*6.31*	*0.00002*	*0.77188*	*0.5958*
2	*GMCSF*	*0.38789*	*6.05*	*0.00005*	*0.75842*	*0.5752*
3	*TNFa*	*0.96889*	*5.90*	*0.00007*	*0.75020*	*0.5628*
4	*IL6*	*0.00182*	*5.64*	*0.00013*	*0.73519*	*0.5405*
5	*Mip1B*	*0.44855*	*5.09*	*0.00052*	*0.70000*	*0.4900*
6	*IFNa2*	*0.13301*	*3.77*	*0.01683*	*0.58737*	*0.3450*
7	*MCP1*	*0.00170*	*3.61*	*0.02400*	*0.57105*	*0.3261*
8	*GCSF*	*0.00315*	*3.45*	*0.03440*	*0.55353*	*0.3064*
9	*IL2*	*0.90769*	*3.29*	*0.04878*	*0.53526*	*0.2865*
10	VEGF	0.03220	3.22	0.05451	0.52726	0.2780
11	Mip1a	0.03774	3.18	0.05710	0.52211	0.2726
12	IL8	0.00086329	3.09	0.06611	0.51166	0.2618
13	EGF	0.33859	3.01	0.07600	0.50100	0.2510
14	TNFB	0.31614	2.76	0.12569	0.46893	0.2199
15	IL4	0.13164	2.64	0.15080	0.45332	0.2055
16	IL10	0.03301	2.56	0.16652	0.44181	0.1952
17	IL7	0.22821	2.35	0.23334	0.41231	0.1700
18	IFNg	0.28550	2.29	0.24001	0.40336	0.1627
19	IL5	1.07906	2.00	0.36824	0.35903	0.1289
20	IL_12p40	0.15047	1.93	0.37360	0.34756	0.1208
21	IL13	0.47673	1.69	0.44061	0.30984	0.0960
22	IL15	0.40696	1.56	0.44061	0.28792	0.0829
23	IL17A	0.32704	1.75	0.44061	0.31843	0.1014
24	IL_12p70	0.21885	1.51	0.44061	0.27875	0.0777
25	IL1a	− 0.03150	− 0.40	0.90517	0.07681	0.0059
26	IP10	− 0.00101	− 0.06	0.95183	0.01000	0.0001

Italic values indicate significance of p < 0.05

*Nine significant associations between cytokines and MK2 were identified (total cytokines 26)

**Sidak correction for p-values is used

**Table 3:** Significant cytokines identified in 29 subjects (15 with Metastasis and 14 without Metastasis)

	Analytes*	Without metastasis, pg/ml (n = 15) Median (Q_1_, Q_3_)	With metastasis, pg/ml (n = 15) Median (Q_1_, Q_3_)	Sidak adjusted p-value**	Effect size
1	*GCSF*	*50.8 (38.5–93.2)*	*1612.4 (894.9–2990.8)*	*0.00012*	*∞*
2	*IL6*	*148.2 (16.1–366.7)*	*5021.7 (3832.1–6510.8)*	*0.00014*	*209*
3	*IL8*	*314.1 (27.1–1758.8)*	*8517.5 (6815.4–11095.9)*	*0.00014*	*209*
4	*MCP1*	*274.9 (158.5–700.5)*	*3348.5 (2093.3–6191.7)*	*0.00064*	*22.3*
5	*IL10*	*7.4 (6.3–10.6)*	*103 (18.3–209.7)*	*0.00245*	*11.7*
6	*TNFa*	*4.1 (3.4–6)*	*8.8 (6.6–15.4)*	*0.00430*	*9.5*
7	GMCSF	8.8 (6.5–12.4)	28.6 (13.5–34.4)	0.05044	4.8
8	Mip1a	28.4 (9.5–49.6)	63.1 (29.8–267.5)	0.08302	4.3
9	IL1B	5.9 (3.9–7.5)	10.9 (6.4–18.1)	0.28424	3.1
10	Mip1B	12.9 (9.8–15.6)	21.2 (13.9–35.2)	0.32911	3.0
11	VEGF	110.8 (44.3–140)	222.3 (80.8–307.5)	0.32911	2.9
12	IL7	4.6 (1–9.1)	17.1 (1–24.8)	0.57380	2.4
13	IL2	4 (1.5–4.9)	7.5 (1.3–11.8)	0.70908	2.2
14	IL4	9.8 (7.1–11.7)	18.9 (0.6–42.7)	0.74789	2.1
15	IL_12p40	12.1 (6.5–14.6)	28.4 (2.8–38.8)	0.75598	2.1
16	IFNa2	21.9 (9.8–34.8)	55.9 (4.6–82.7)	0.77369	2.0
17	IL17A	6.7 (5.2–8.7)	12.8 (5.4–17.5)	0.80220	1.9
18	TNFB	8.4 (5.5–12.4)	15 (1.5–24.2)	0.87285	1.8
19	IFNg	9.8 (7.3–10.6)	16.3 (0.4–25.4)	0.93782	1.6
20	EGF	12.8 (7.5–16.1)	14.9 (6.3–25.9)	0.96572	1.5
21	IL13	7.1 (4.8–10.4)	9.8 (2.4–13.8)	0.98874	1.3
22	IL_12p70	7.8 (7.2–9.8)	10.4 (2.1–17.4)	0.98874	1.3
23	IL1a	7 (6.5–9.2)	8.2 (2.7–13.1)	0.99124	1.2
24	IL15	10.2 (7.9–14.5)	10.2 (3.8–14.1)	0.99124	1.2
25	IL5	4.4 (3.7–5.5)	5.4 (0.7–6.7)	0.99124	1.2
26	IP10	41.9 (26.1–66.7)	41 (30–113)	0.99124	1.1

Italic values indicate significance of p < 0.05

*Six significant cytokines identified (total cytokines 26)

**Sidak correction for multiple comparisons for the Kruskal–Wallis test

### Cytokine networking linked with metastasis in gastric cancer

**Fig. 5:**
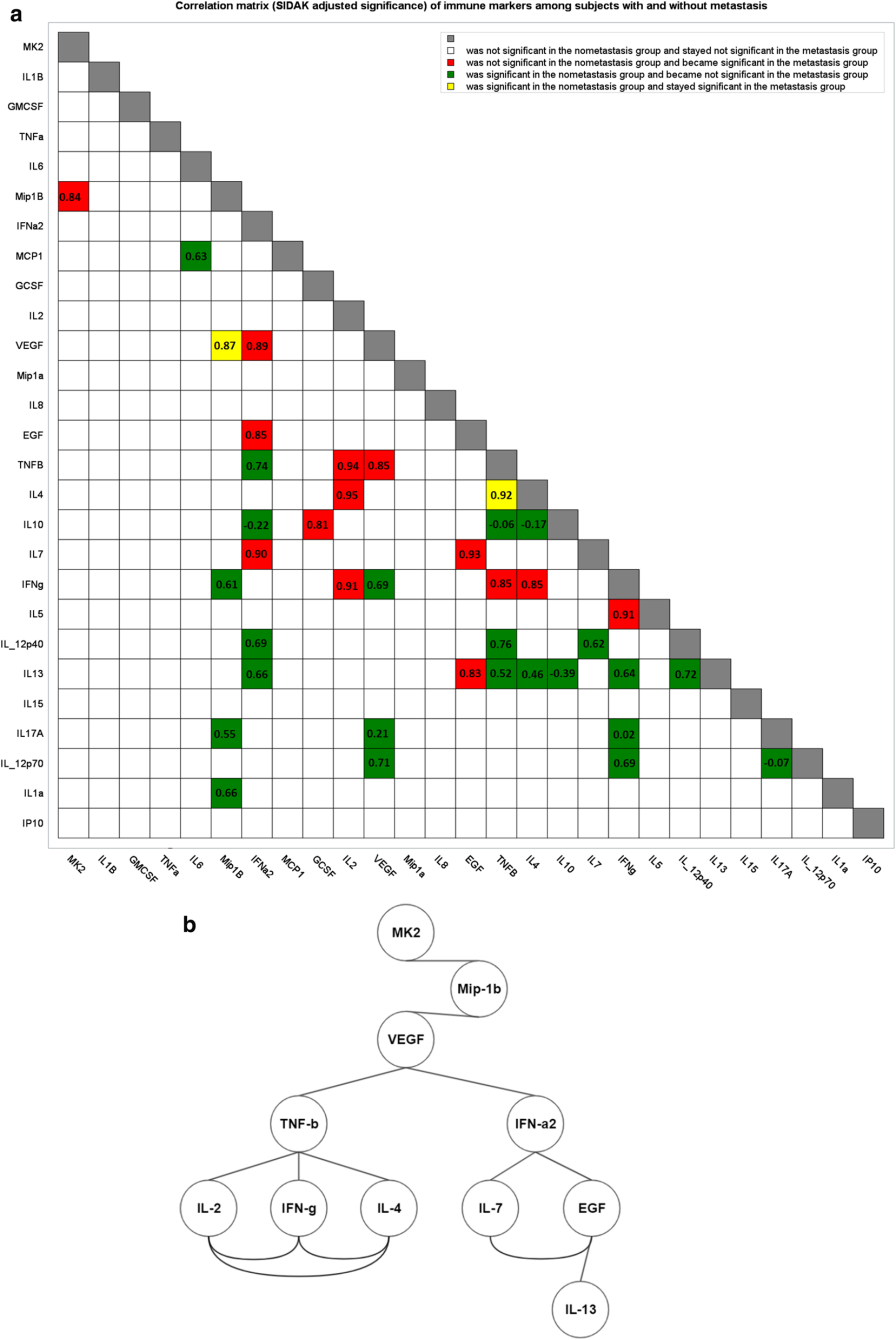
**a** A lower triangular correlation matrix in which coloring is used to highlight the Spearman correlation’s Sidak adjusted significance of the p-value between subjects with and without metastasis for MK2 and every cytokine with all other cytokines. **b** A rooted correlation network at MK2 of cytokines and chemokines among gastric metastasized subjects. The 1st chain of correlation consists of Mip-1β. The 2nd chain of correlation consists of VEGF. The 3rd chain of correlation consists of TNF-β and IFN-α2. This correlation network reflects the significant interrelationship between MK2 and cytokines/chemokines (red and yellow colored boxes in the SIDAK adjusted correlation matrix)

**Fig. 6:**
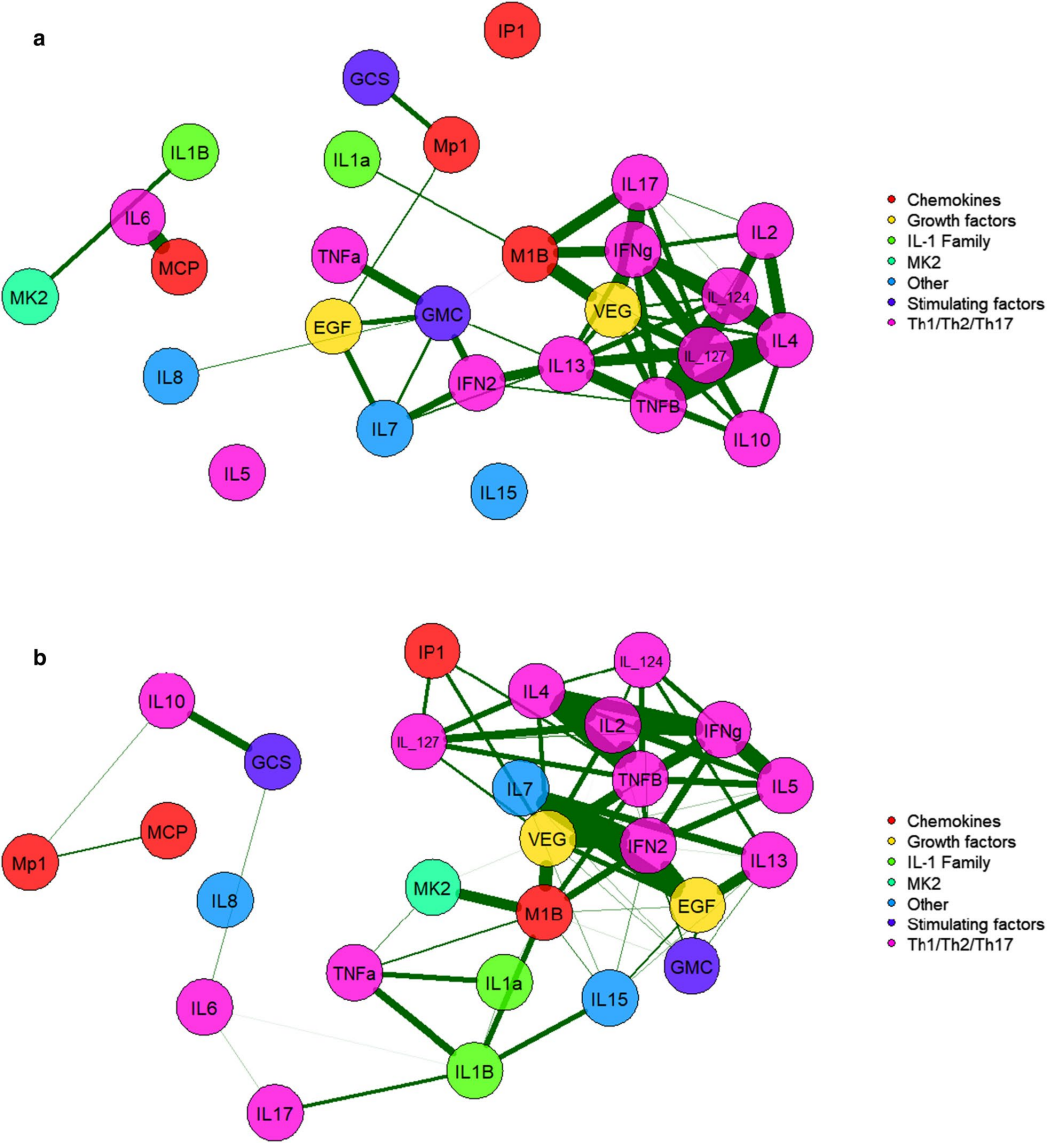
**a** Intercorrelation between MK2, cytokines, chemokines, growth factors and stimulating factors within the non-metastasis group. **b** Intercorrelation between MK2, cytokines, chemokines, growth factors and stimulating factors within the metastasis group. In both figures we only keep spearman correlation of 0.65 or more

To better understand the difference between metastasized and non-metastasized subjects, we present cytokines and MK2 jointly in a correlation network as shown in Fig. 5a. Specifically, (i) green indicates that markers were significant in the non-metastasis group and became not significant in the metastasis group; (ii) red indicates that markers were not significant in the non-metastasis group and became significant in the metastasis group; (iii) white indicates that markers were not significant in the non-metastasis group and stayed not significant in the metastasis group; and (iv) yellow indicates that markers were significant in the non-metastasis group and stayed significant in the metastasis group. Figure 5a also indicates that the significant association between IL-6 and MCP-1 diminishes among metastasis subjects. Notably, TNF-α and IL-1β were significantly associated with each other among subjects with metastasis, but such significance became marginal after the Sidak adjustment and hence their association is not present in Fig. 5a. In Fig. 5a, b rooted correlation network is shown, suggesting some previously unreported associations between MK2, Mip-1β, and T cell related cytokines. To give a comprehensive pictorial representation of the intercorrelation between MK2, cytokines, chemokines, growth factors and stimulating factors, while emphasizing the clinical significance instead of statistical significance only, we provide Fig. 6a, b. While Fig. 5 illustrates the significant association of cytokines/chemokines with metastasis, Fig. 6 illustrates associations that may not be statically significant but show clinically relevant effect size noting that the thickness of a line connection cytokines/chemokines indicates the magnitude of the spearman correlation. Figure 6a, among patients without metastasis, reveals lack of any meaningful association between MK2 with Type 1 and Type 2 helper T cells, but Fig. 6b, among patients with metastasis, demonstrates how MK2 gains association with T cells via a strong correlation between MK2 and Mip-1β.

## Discussion

Since gastric cancer is strongly associated with inflammation, but is also a more difficult cancer to treat, continued studies of mechanisms regulating inflammation are needed to develop novel treatment approaches. This study is the first to demonstrate how the MK2 pathway is linked to cytokine production and metastasis in gastric cancer. MK2 expression was found to be associated with known downstream cytokines IL-1β, IL-6, and TNF-α, but also some previously unrecognized associations with GM-CSF, Mip-1β, IFN-α2, MCP-1, G-CSF, and IL-2. It is noteworthy that G-CSF had the strongest association with metastasis, which is in agreement with our previous work showing increased gene expression in gastric tumors [18]. Another group recently confirmed these findings in gastric cancer [19] and there has been some data published that G-CSF is highly expressed in breast cancer [20, 21].

While MK2 was found to be significantly associated with a long list of cytokines and chemokines amongst gastric cancer patients including IL1-β, GM-CSF, TNF-a, IL-6, Mip-1β, IFN-α2, MCP-1, G-CSF, and IL-2; it was only significantly associated, after the Sidak correction for multiple testing, with Mip-1β among those who metastasized, revealing a new link to gastric metastasis. Studies by other groups have suggested pro-metastatic activity of Mip-1β [22, 23], but there has also been suggestions otherwise [24]. Thus, more in depth mechanistic studies are needed to assess the overall impact of this chemokine in cancer.

Our recent study using mice has shown that MK2 indeed regulates macrophage chemokine activity (MCP-1, Mip-1α, and Mip-2α) and recruitment to promote colon tumor growth [13]; however this is the first study to show an association between MK2 and Mip-1β. Although the later study didn’t address the connection between MK2 and Mip-1β in particular, MIP-1α and MIP-1β although distinct, they are nonetheless highly homologous chemokines [25]. These results, our previous mouse study, and recent studies by other group have begun to highlight the importance of the MK2 pathway in regulating macrophage inflammatory cytokine/chemokine production in tumors, infection, and other injury states [13, 26, 27]. While the found role of Mip-1β in connecting MK2 pathway to Th1 and Th2 pathways associated with VEGF in our study is surprising, it’s not unforeseen given that the chemokines could be associated with Th1 and Th2 responses [28, 29]. Another group also suggested that Mip-1β induced VEGF expression in oral cancer supporting our findings that there may be a link between these two factors [30]. The use of intercorrelation networks to identify new links such as the link between MK2 and Mip-1β at the metastasis state is novel. Such intercorrelation networks could reveal both associations and the drivers of associations that one could not discover otherwise when using the standard mean concentrations comparisons. Specifically, in here, the rooted correlation network was able to provide an idea about the mechanism behind the association between MK2 and Mip-1β at the metastasis state by connecting MK2 pathway to Th1 and Th2 pathways associated with VEGF. Such discovery builds upon, but is not warranted with the classical rooted correlation network [16].

Independent of cytokines and chemokines, MK2 alone was able to correctly classify gastric cancer patients by metastasis status 85.7% of the time. In fact an optimal cut-off of 4.84 for MK2 was found to give a good accuracy as a diagnostic test for metastasis (AUC = 0.83) with both high sensitivity and specificity (Sn = 0.87; and Sp = 0.79 respectively), while noting that the median of MK2 among metastasized subjects was 12.81 relative to only 3.36 among non-metastasized subjects (p-value = 0.001). Data in mice have shown that MK2 does contribute to tumor progression by promoting M2 macrophage polarization and tumor angiogenesis which in turns promotes tissue renovation that governs cell invasion and metastasis [31]. Furthermore, inhibition of the stromal p38MAPK/MK2 pathway was found to limit breast cancer metastases in mice [32]. These data from others along with our data showing the striking classification of metastasis status among gastric cancer patients by MK2 and its association with a wide range of cytokines suggests pathway blockade is promising for immunotherapy treatment [33].

Our analysis with Sidak correction for multiple testing, showed significant elevation of G-CSF, IL-6, IL-8, MCP-1, IL-10, TNF-α, GM-CSF, and Mip-1α in metastasized subjects compared to non-metastasized ones with GCSF being the most significant biomarker. Of a great revelation is the strength of association between G-CSF and IL-10, which was found to be striking among those who metastasized compared to those who did not as illustrates in Additional file 1: Figure S1. In fact, the ranges of IL-10 and G-CSF values among none metastasized subjects were from 4.4 to 25.3 and 3.9 to 234.8, respectively, but substantially higher among metastasized subjects as it went from 7.6 to 321.5 and 635.3 to 3998.3, respectively. While it was shown that G-CSF is highly expressed in human gastric and colon cancers and promote carcinoma cell proliferation and migration [18], its strong association with IL-10 upon metastasis among gastric cancer patients has not been explored until this work. In other types of cancer, it was shown that indeed such strong association does exist in a pairwise correlations between the cytokine production levels in a culture supernatant of biopsy samples of mammary adenocarcinoma, however; data were lumped together from patients without and with metastases in regional lymph nodes giving a correlation of r(IL-10, G-CSF) = 0.61 and p-value = 0.0012 [34]. We showed a more general role for G-CSF in IL-10 production in our previous study [17]. Thus, this cytokine should be examined further as a biomarker.

## Conclusions

We are the first to show the significance of the MK2 pathway in gastric cancer. Of particular interest is that increased gene expression is strongly associated with metastasis. Gene expression was able to classify gastric cancer metastasis 85.7% of the time. Thus, MK2 is a potential biomarker for gastric cancer metastasis. Furthermore, MK2 expression was associated with a panel of cytokines, some of which are known downstream mediators, such as MCP-1, IL-1β, IL-6, and TNF-α. Others, such as G-CSF, GM-CSF, Mip-1β and IFN-α are previously unrecognized in associated with MK2. We found G-CSF to have the strongest association with metastasis with the lowest p-value. Furthermore, Mip-1β showed significant correlation with MK2 and metastasis after Sidak correction. Thus, MK2 and associated cytokines/chemokines are potential biomarkers for gastric cancer metastasis. The intercorrelation analysis approaches are a promising tool to more clearly understand the complexities of cytokine/chemokine regulation and association with disease outcome.

## Supplementary information

**Additional file 1: Figure S1.** The association between GCSF and IL-10 stratified by metastasis status.

## Data Availability

Molecular Biology reagents were purchased from ThermoFisher Scientific Cytokine Arrays were purchased from EMD Millipore

## Abbreviations

MK2: Map kinase-activated protein kinase 2
OR: Odds ratio
ROC: Receiver operator characteristic
AUC: Area under curve
qRT: Quantitative real time
