# Supplementary figures and images for: The MK2 pathway is linked to G-CSF, cytokine production and metastasis in gastric cancer: a novel intercorrelation analysis approach
Source: J Transl Med. 2020 Mar 26;18:137. doi: 10.1186/s12967-020-02294-z (PMC7098132; doi:10.1186/s12967-020-02294-z)

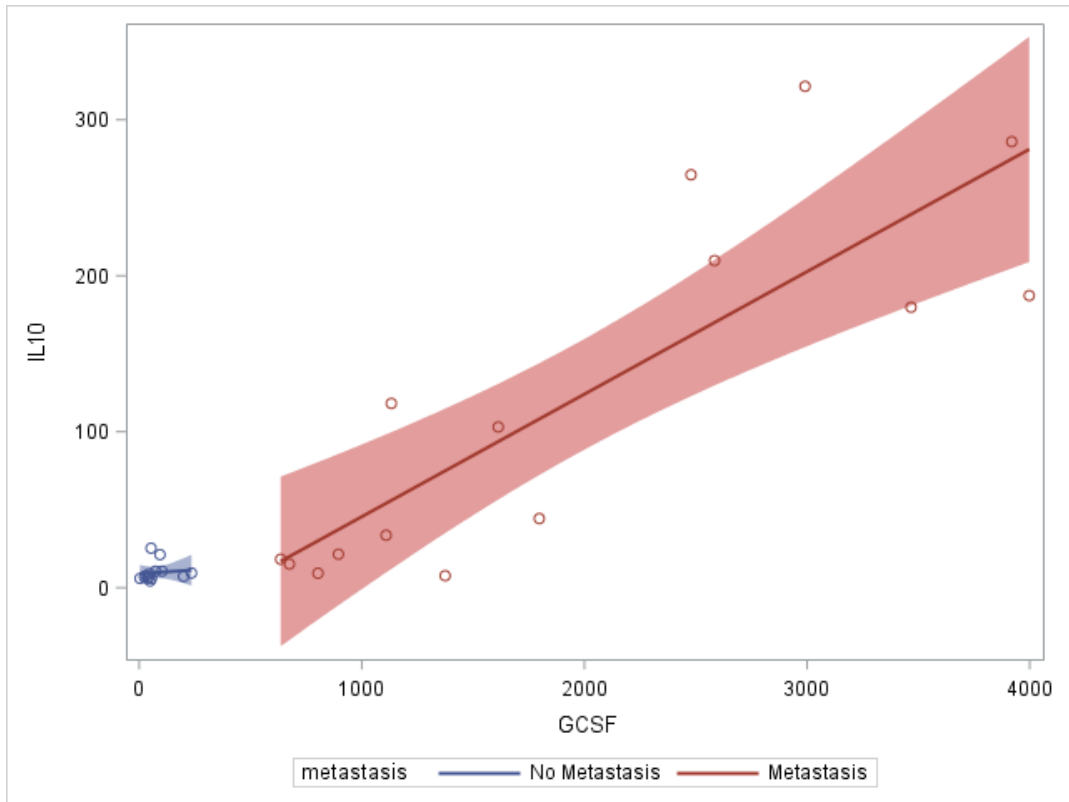

**Figure S1:** The association between GCSF and IL-10 stratified by metastasis status.

Supplement: Supplementary file 1 — Additional file 1: Figure S1. The association between GCSF and IL-10 stratified by metastasis status. [file 12967_2020_2294_MOESM1_ESM.pdf]
